# Physiological and Transcriptome Analyses of Early Leaf Senescence for *ospls1* Mutant Rice (*Oryza sativa* L.) during the Grain-Filling Stage

**DOI:** 10.3390/ijms20051098

**Published:** 2019-03-04

**Authors:** Zhaowei Li, Xinfeng Pan, Xiaodong Guo, Kai Fan, Wenxiong Lin

**Affiliations:** 1 College of Life Sciences, Fujian Agriculture and Forestry University, Fuzhou 350002, China; zhaoweili@fafu.edu.cn; 2 Fujian Provincial Key Laboratory of Agroecological Processing and Safety Monitoring, Fujian Agriculture and Forestry University, Fuzhou 350002, China; 3165006035@m.fafu.edu.cn (X.P.); 3157614046@m.fafu.edu.cn (X.G.); fankai@fafu.edu.cn (K.F.); 3 College of Crop Science, Fujian Agriculture and Forestry University, Fuzhou 350002, China

**Keywords:** rice (*Oryza sativa* L.), *OsVHA-A*, leaf senescence, RNA-seq, hormones

## Abstract

Early leaf senescence is an important agronomic trait that affects crop yield and quality. To understand the molecular mechanism of early leaf senescence, *Oryza sativa premature leaf senescence 1* (*ospls1*) mutant rice with a deletion of *OsVHA-A* and its wild type were employed in this study. The genotype-dependent differences in photosynthetic indexes, senescence-related physiological parameters, and yield characters were investigated during the grain-filling stage. Moreover, RNA sequencing (RNA-seq) was performed to determine the genotype differences in transcriptome during the grain-filling stage. Results showed that the *ospls1* mutant underwent significant decreases in the maximal quantum yield of photosystem II (PSII) photochemistry (Fv/Fm), net photosynthesis rate (*Pn*), and soluble sugar and protein, followed by the decreases in *OsVHA-A* transcript and vacuolar H^+^-ATPase activity. Finally, yield traits were severely suppressed in the *ospls1* mutant. RNA-seq results showed that 4827 differentially expressed genes (DEGs) were identified in *ospls1* mutant between 0 day and 14 days, and the pathways of biosynthesis of secondary metabolites, carbon fixation in photosynthetic organisms, and photosynthesis were downregulated in the senescing leaves of *ospls1* mutant during the grain-filling stage. In addition, 81 differentially expressed TFs were identified to be involved in leaf senescence. Eleven DEGs related to hormone signaling pathways were significantly enriched in auxin, cytokinins, brassinosteroids, and abscisic acid pathways, indicating that hormone signaling pathways participated in leaf senescence. Some antioxidative and carbohydrate metabolism-related genes were detected to be differentially expressed in the senescing leaves of *ospls1* mutant, suggesting that these genes probably play response and regulatory roles in leaf senescence.

## 1. Introduction

Senescence is the final stage of leaf development and corresponds to the programmed degradation of cells, tissues, organs, and the entire organism [1]. Leaf senescence is not a passive and confused deterioration process. The senescing leaves undergo dramatically coordinated changes at the physiological, biochemical, and molecular levels during the senescence period [2]. For example, the expression of senescence-associated genes (SAGs), chlorophyll breakdown, photosynthesis termination, membrane deterioration, malondialdehyde (MDA) accumulation, and macromolecule hydrolysis comprise the senescence physiological processes, which cause the disassembly of cell structure and cell death [1,3]. While leaf senescence is a deleterious process, it has a special role in recruiting nutrients from senescing tissues to still living parts of the plant. In the senescing leaves of annual plants, the hydrolysates of macromolecules, such as starch, proteins, lipids, and nucleic acids, are trans-located to developing seeds and fruits and contribute to the final yields. Thus, reasonable leaf senescence is essential to maximize viability during the plant’s life cycle [4]. However, early leaf senescence often occurs when plants encounter various stresses. Early leaf senescence will shorten the growth phase of crops and result in losses of yield and quality, which are extremely harmful to agronomic production. Until now, early leaf senescence has become an increasing concern in pace with global climate changes in recent decades [5].

Rice (*Oryza sativa* L.) is one of the most important cereal crops and serves as a staple food that feeds more than a half of the world’s population, mainly in Asia [6]. In rice, grain yield is greatly dependent on photosynthetic substances of functional leaves during the grain-filling stage, and extending leaf green and photosynthesis duration is crucial to obtaining superior yield. However, leaf senescence often occurs too early under severe environmental stresses, and an early occurrence of leaf senescence caused by adverse environmental stresses leads to a drop in photosynthesis and precocious cell death [7,8]. Early leaf senescence during the grain-filling stage retards the translocation of nutrients from source leaves to developing grains, resulting in incomplete grain setting and reduced final yield [9]. Over the past few decades, many advances have been achieved in understanding the yield effects, onset and progression, and genetic control of early leaf senescence at the physiological and molecular levels [8,10,11]. The most distinguishing characteristics of early leaf senescence in rice are chloroplast degeneration and chlorophyll degradation, followed by the accumulation of reactive oxygen species (ROS) such as superoxide anion radicals (O_2_^−^), hydroxyl radicals (OH∙), singlet oxygen (^1^O_2_), and hydrogen peroxide (H_2_O_2_). ROS, as signaling molecules, affect the expression of SAGs, thereby resulting in oxidative stress and damage to cellular organelles [12,13]. Meanwhile, a large number of SAGs have been experimentally identified in rice, including numerous transcription factors (TFs), such as *WRKY*, *NAC*, *MYB*, and *bZIP* family members; transporters; defense-related genes; signal transduction-related proteins [8]; kinases and receptor-like kinases; and regulators of metabolism [11]. Moreover, leaf senescence of rice is accelerated by abscisic acid (ABA), brassinosteroids (BRs), ethylene (ET), and methyl jasmonate [14]. Exogenously applied ABA and salinity induce the expressions of several SAGs to accelerate leaf senescence, indicating the substantive mechanisms of connection between leaf senescence, ABA, and stress signaling [15,16]. Early leaf senescence in rice is an integrated metabolic response to various stimulations, dominated by extremely complex transcriptional regulatory networks.

Vacuolar H^+^-ATPase (V-H^+^-ATPase) is a large multi-heteromeric protein complex making up 6.5%–35% of the total tonoplast protein mass and is of prime importance for plant development and stress adaptation [17,18]. This protein complex is mainly located in vacuolar membranes, plasma membranes, and other endomembrane systems. It pumps protons into membrane-surrounded intracellular compartments at the expense of hydrolysis energy of ATP and is required to activate secondary transport processes across tonoplast and vesicle dynamics [19,20]. In plants, V-H^+^-ATPase has been proved to be indispensable in several cellular processes and physiological responses, including membrane trafficking, male gametophyte development [21], lateral root development, stomatal density and opening [22], environmental stress tolerance [23], leaf senescence, and seed dormancy [24]. Biochemical experiments on the subunit composition of V-H^+^-ATPase have showed that V-H^+^-ATPase is built from up to 14 subunits, among which the V-H^+^-ATPase subunit A (VHA-A) is an indispensable catalytic subunit protruding into the cytoplasm [20]. In *Arabidopsis*, the deficiency of *VHA-A* transcripts damages complete male and partial female gametophytes, owing to abnormal morphological changes in Golgi stacks and Golgi-derived vesicles [21]. In rice, RNAi-mediated inhibition of *OsVHA-A* transcriptions increases stomatal density and aperture, thereby increasing the susceptibility to drought and salt stress [22]. Yang et al. [24] revealed that a decrease in *OsVHA-A* transcription for *Oryza sativa premature leaf senescence 1* (*ospls1*) mutant rice gave rise to ROS accumulation and salicylic acid (SA) increase, resulting in a phenotype of early leaf senescence. In a word, these studies support the crucial role of *OsVHA-A* in rice plant growth, development, and senescence. However, until now, the molecular mechanism and global transcriptional control on the involvement of *OsVHA-A* in early leaf senescence in rice remain poorly understood.

In this study, a premature senescence rice mutant, named *ospls1*, and its corresponding wild type were employed to compare the genotype-dependent differences in agronomic characteristics, photosynthetic ability, and senescence-related physiological parameters of leaves during the grain-filling stage. Meanwhile, the expression of the mutant gene, *OsVHA-A*, which caused the phenotype of premature leaf senescence, was detected for two rice genotypes by quantitative real-time polymerase chain reaction (qRT-PCR). Based on the revolutionary technological superiority in understanding complex eukaryote transcriptome, RNA sequencing (RNA-seq) was used to investigate the transcriptome differences in flag leaves of *ospls1* mutant rice and its wild type during the grain-filling stage. According to extensive data analyses, many differentially expressed genes (DEGs) and metabolic pathways were identified and characterized, which were involved in early leaf senescence in terms of *OsVHA-A* mutation in *ospls1* mutant rice during the grain-filling stage.

## 2. Results

### 2.1. Characterization of Phenotype, Major Agronomic Traits, and Biochemical Changes of ospls1 Mutant during the Grain-Filling Stage

**Figure 1:**
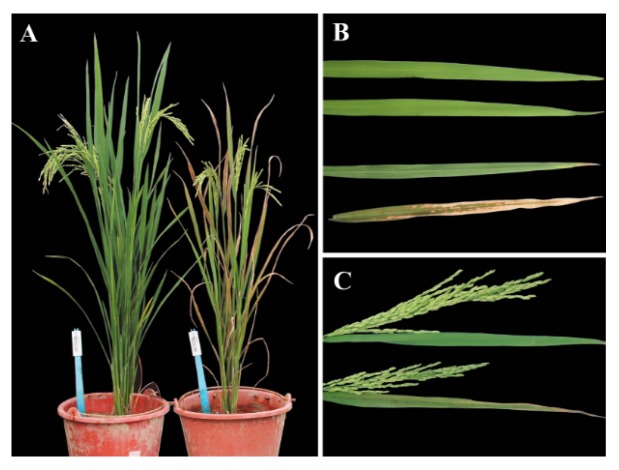
Comparison of phenotypes between the *ospls1* mutant and wild type. (**A**) Plant morphological phenotype during the grain-filling period: left designates the wild type, and right designates the *ospls1* mutant; and (**B**) flag leaves of the two genotypes. The upper two leaves represent the wild type at day 0 and 14 of grain-filling stage; the lower two leaves represent the *ospls1* mutant at day 0 and 14 of grain-filling stage; (**C**) spikes with flag leaves on day 7 of the grain-filling stage; the upper indicates the wild type, and the lower indicates the *ospls1* mutant.

At the heading stage, the lower leaves of *ospls1* mutant plant showed visible early senescence symptoms, and flag leaves retained normal green appearance (Figure 1A). After heading, the flag leaves of *ospls1* mutant appeared to exhibit early senescence symptoms and became obviously visible on the 14th day of grain filling, followed by reddish brown lesions extending to the leaf blade (Figure 1B). The *ospls1* mutant presented a shorter panicle than the wild type (Figure 1C). In addition, the *ospls1* mutant also displayed significant differences from wild type in terms of plant height, available panicle number, seed-setting rate, 1000-grain weight, yield per plant, and harvest index (Table 1). Under field condition, the plant height of *ospls1* mutant was 92.4 cm (2015), which was significantly shorter than that of the wild type, 109.3 cm (*p* < 0.05, 2015). Available panicle numbers of the *ospls1* mutant and wild type were 6.3 and 11.7, respectively. The seed-setting rate and 1000-grain weight of the *ospls1* mutant were 42.8% and 17.62 g, respectively, which were significantly less than those of the wild type, 84.5% and 23.27 g (*p* < 0.05, 2015). In comparison with the wild type, the *ospls1* mutant exhibited remarkable early leaf senescence and decreases in yield and harvest index.

**Figure 2:**
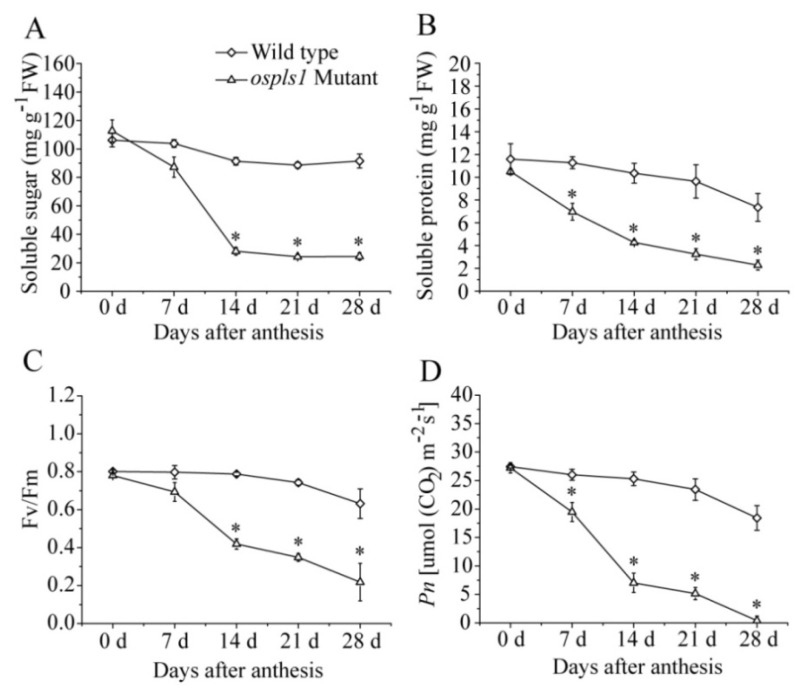
Temporal patterns of soluble sugar (**A**), soluble protein (**B**), Fv/Fm (**C**), and *Pn* (**D**) in flag leaves of the two rice genotypes during the grain-filling stage. Vertical bars represent standard errors with three independent biological replications. The asterisks represent significant differences (*p* < 0.05).

In this study, soluble sugar, soluble protein, maximal quantum yield of PSII photochemistry (Fv/Fm), and net photosynthesis rate (*Pn*) were determined to compare the senescence-associated changes in the flag leaves of the two rice genotypes during the grain-filling stage. As shown in Figure 2, the *ospls1* mutant showed a rapid decrease in the soluble sugar and protein at the initial stage of grain filling and reached minimum levels on day 14 of grain filling. This phenomenon was not observed in the wild type, which presented relatively stable soluble sugar levels and a slight decrease in soluble protein during the grain-filling stage (Figure 2A,B). In addition, Fv/Fm and *Pn* in the leaves of *ospls1* mutant also sharply decreased and were significantly lower than those in the wild type over the entire grain-filling stage (Figure 2C,D), suggesting a weak light-harvesting and photosynthetic ability for *ospls1* mutant rice.

### 2.2. Differential Expressions of OsVHA-A and V-H^+^-ATPase Activities in ospls1 Mutant and Wild Type

**Figure 3:**
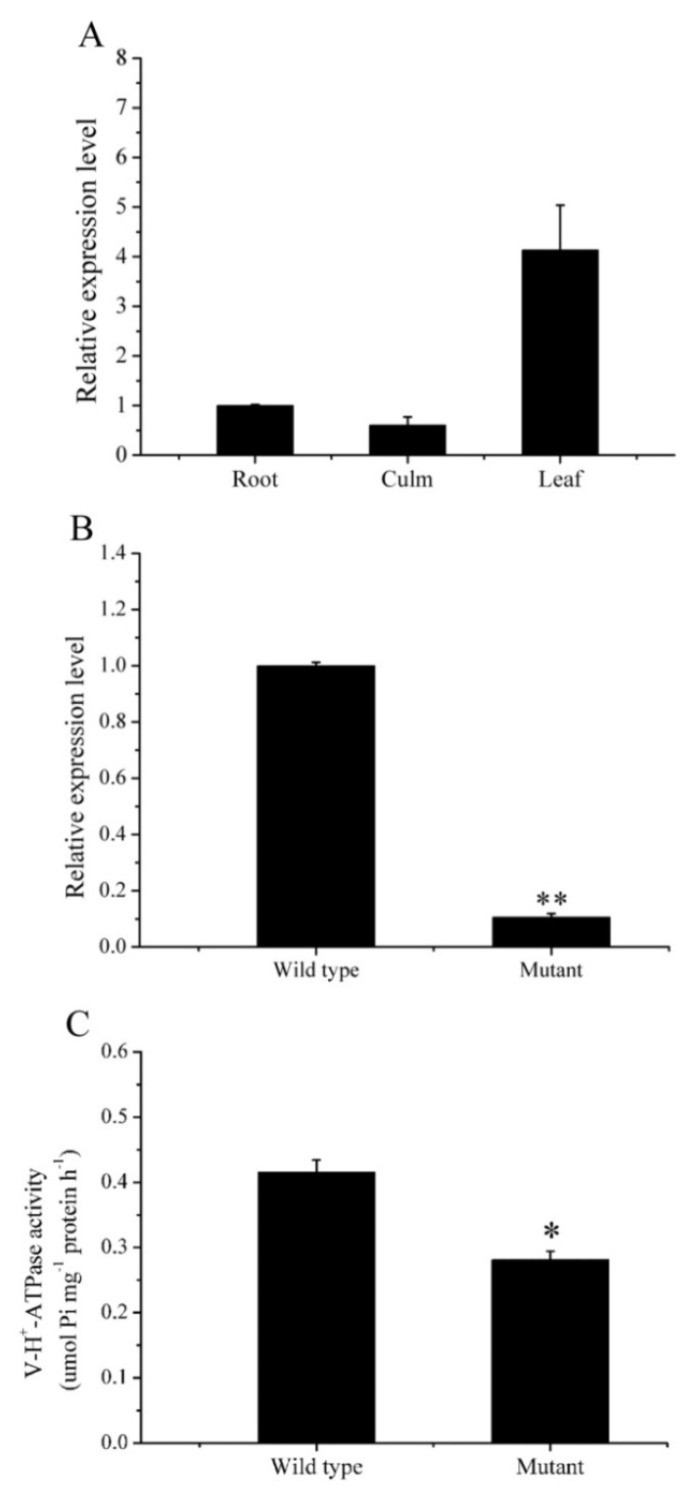
Analyses of *OsVHA-A* expression and V-H^+^-ATPase activity in various tissues and genotypes. (**A**) Expression levels of *OsVHA-A* in root, culm, and leaf; and (**B**) genotype-dependent expression levels of *OsVHA-A* in the flag leaves at the heading stage. (**C**) V-H^+^-ATPase activities in the flag leaves of the two rice genotypes at the heading stage; vertical bars represent standard errors with three independent biological replications. * and ** represent significant differences at 0.05 and 0.01 probability levels, respectively.

As shown in Figure 3, the expression patterns of *OsVHA-A* were remarkably dependent on tissues and genotypes. *OsVHA-A* was expressed in the leaf blade of rice, rather than the root or culm (Figure 3A). By contrast, the *ospls1* mutant showed extremely low expression levels of *OsVHA-A* in flag leaves than the wild type, which held a large number of *OsVHA-A* transcripts in the same tissue (Figure 3B). As expected, V-H^+^-ATPase activity in flag leaves of *ospls1* mutant was significantly lower than that in wild type (Figure 3C). These results suggested the deficiency in *OsVHA-A* transcript and decrease in V-H^+^-ATPase activity for *ospls1* mutant rice.

### 2.3. Evaluation of RNA-Seq Reads and Mapping Results

To obtain an overview of the differences in transcriptome between *ospls1* mutant and wild type, RNA was extracted individually from 12 flag leaf samples, and double-stranded cDNA was synthesized. After ligating adapter sequences, 12 ds-cDNA libraries were constructed by PCR reaction. The cDNA libraries of flag leaves were sequenced with three biological replicates using the Illumina deep-sequencing technique. A total of 45–62 million raw reads were generated from each cDNA library. Clean reads were obtained after filtering out low-quantity sequences. Approximately 80% of clean reads were mapped to the reference genome, among which nearly 77% were mapped to unique locations (Table 2). Meanwhile, 89,988 genes were aligned for the tested samples, thus providing massive data for further analyses on expression profiling and metabolism pathways.

### 2.4. Identification of Differentially Expressed Genes (DEGs) and Confirmation of Tag-Mapped Genes by Quantitative Real-Time Polymerase Chain Reaction (qRT-PCR)

**Figure 4:**
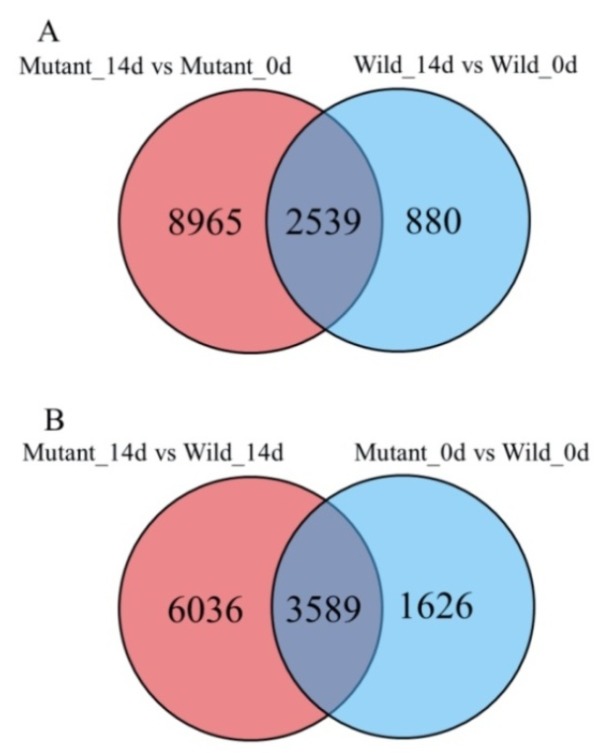
Venn diagrams of DEGs in different comparisons. The numbers indicate unique and common DEGs in different comparisons. (**A**) Unique and common DEGs between Mutant_14d vs. Mutant_0d and Wild_14d vs. Wild_0d; and (**B**) unique and common DEGs between Mutant_14d vs. Wild_14d and Mutant_0d vs. Wild_0d.

The fragments per kilobase of transcript sequence per million base pairs sequenced (FPKM) method was employed to normalize gene expression counts for the sequence. The DEGs were identified using the DESeq software package, according to padj < 0.05. As shown in Table 3, 11,504, 3419, 5215, and 9625 DEGs were identified in mutants between 0 day and 14 days, wild type between 0 day and 14 days, wild type and mutant at 0 day, and wild type and mutant at 14 days, respectively. The greatest expression differences were found in mutant between 0 day and 14 days, indicating that the *ospls1* mutant held more SAGs during the senescing stage. Among them, 5312 DEGs were found to be upregulated, and 6192 DEGs were downregulated. The smallest expression differences were found in wild type between 0 day and 14 days, and 1636 DEGs were upregulated, whereas 1783 DEGs were downregulated. By contrast, 9845 unique and 2539 common DEGs were identified by comparing the two libraries, including 8965 unique DEGs from mutant between 0 day and 14 days and 880 unique DEGs from wild type between 0 day and 14 days (Figure 4A). In terms of genotypes, a total of 5215 and 9625 DEGs were identified between mutant and wild type at 0 day and 14 days, including 3093 upregulated genes and 2122 downregulated genes in mutant and wild type at 0 day, and nearly equal proportion between up and downregulated genes in two genotypes at 14 days (Table 3). In addition, 1626 and 6036 unique DEGs were identified in mutant and wild type at 0 day and 14 days, whereas 3589 DEGs were overlapped between the two libraries (Figure 4B), suggesting that different subsets of genes underlying different response mechanisms were affected in the flag leaves of *ospls1* mutant and wild type during the grain-filling stage.

To validate the results of RNA-seq data, a subset of 15 DEGs was randomly selected for qRT-PCR analysis. The gene ID and description of DEGs are listed in Supplementary Table S1. As shown in Supplementary Figure S1, the expression profiles of qRT-PCR were highly consistent with the results of RNA-Seq analysis, indicating that the DEG-based technique for counting transcripts gave an accurate reflection of transcript abundance and could be employed in gene expression analysis related to the pathways of early leaf senescence in the *ospls1* mutant.

### 2.5. DEGs Significantly Enriched in Metabolic Pathways for the Two Rice Genotypes

All DEGs of the two rice genotypes were analyzed through Kyoto Encyclopedia of Genes and Genomes (KEGG) Orthology Based Annotation System (KOBAS) to identify the metabolic pathways in which they function. For *ospls1* mutant, 2349 downregulated and 2478 upregulated genes were mapped to 117 KEGG pathways (Supplementary Table S2). Among them, three metabolic pathways were significantly downregulated (corrected *p*-value < 0.05), whereas no pathway was significantly upregulated. By contrast, only 623 downregulated genes in the wild type were mapped to 99 KEGG pathways, and 768 upregulated genes were mapped to 96 KEGG pathways (Supplementary Table S3). Three of these pathways were significantly downregulated, and six metabolic pathways were significantly upregulated (corrected *p*-value < 0.05) during the grain-filling stage (Table 4). These typical senescence symptoms, such as carbon fixation in photosynthetic organisms and photosynthesis, were significantly overrepresented for *ospls1* mutant in the downregulated pathways, whereas those were not observed for wild type. For the *ospls1* mutant, a total of 278 downregulated genes were significantly enriched in the biosynthesis of secondary metabolites; however, for the wild type, only 82 downregulated and 95 upregulated genes were enriched. In addition, two pathways of nitrogen metabolism and carotenoid biosynthesis were significantly enriched in the downregulated genes for wild type. Moreover, certain protective activity pathways were obviously activated in the wild type, such as plant-pathogen interaction, fatty acid degradation, flavonoid biosynthesis, and alpha-linolenic acid metabolism. Meanwhile, amino sugar and nucleotide sugar metabolism was significantly enriched in the unregulated genes for the wild type. These results indicated that distinguishing pathways were involved in the differential leaf development processes of the two rice genotypes during the grain-filling stage.

### 2.6. Transcription Factor (TF) Families in the DEGs of the Two Genotypes

**Figure 5:**
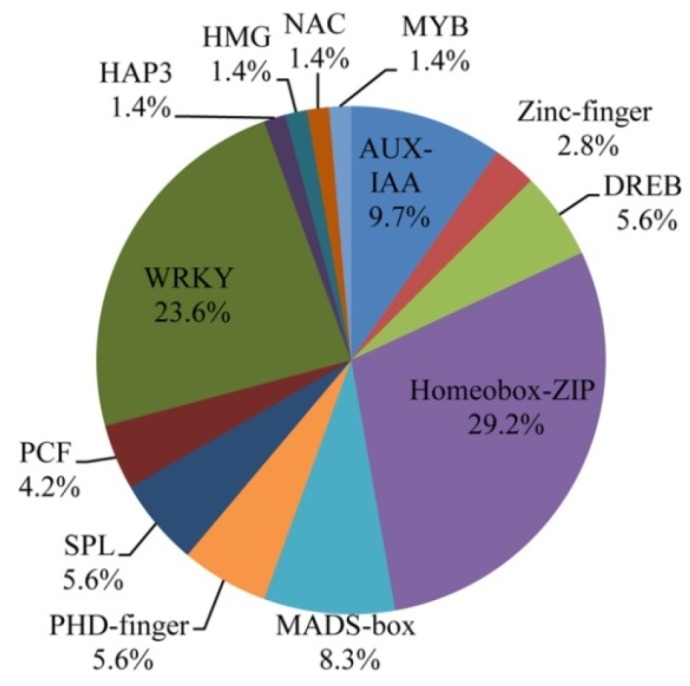
TF families identified in the two rice genotypes. Number of transcripts related to each specific family was clustered and shown as the percentage of the total number of TFs.

**Figure 6:**
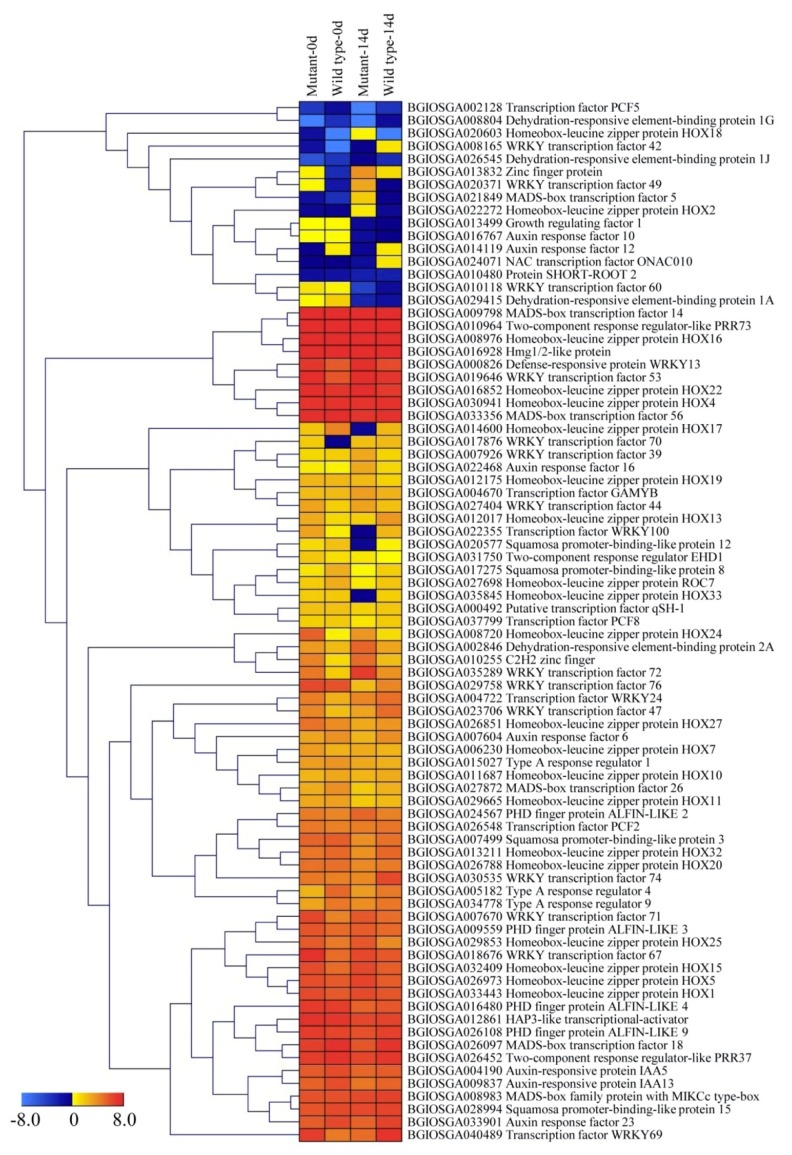
Hierarchical cluster analysis of TFs identified in DEGs in the two rice genotypes. Genes were displayed by different colors. Relative levels of expression were shown by a color gradient from low (blue) to high (red).

In this study, 81 DEGs between 0 day and 14 days in these two genotypes were identified and characterized as TFs (Supplementary Table S4). They were clustered into 13 gene families: *Homeobox-ZIP*, *WRKY*, *AUX-IAA*, *NAC*, *HMG*, *HAP3*, *PCF*, *SPL*, *PHD-finger*, *MADS-box*, *DREB*, *Zinc-finger*, and *MYB* (Figure 5). The most highly represented family was *Homeobox-ZIP* (29.2%). The majority of *Homeobox-ZIP* genes had significantly high transcript abundance in the leaves of the two genotypes, such as *homeobox-leucine zipper protein HOX1*, *HOX4*, *HOX5*, *HOX7*, *HOX10*, *HOX11*, *HOX15*, *HOX16*, *HOX20*, *HOX22*, *HOX27*, and *HOX32* (Figure 6). Another significantly represented family was *WRKY*, around 23.6% of the TFs (Figure 5). The *WRKY TFs 24*, *39*, *44*, *53*, *67*, *70*, *71*, *72*, *74*, and *76* showed steadily high transcript abundance in the two genotypes, whereas *WRKY TFs 42*, *49*, and *70* were among those differentially expressed in the *ospls1* mutant and wild type (Figure 6). In addition, approximately 9.7% of *TFs* belonged to the *AUX-IAA* family (Figure 5). Among them, *auxin response factor 10* was found mainly in the leaves of the two genotypes at the initial stage of grain filling, and *auxin response factor 12* preferably expressed in the wild type, whereas the transcriptions of other *AUX-IAA* members showed no dependence on the genotypes (Figure 6). Around 8.3% of the differentially expressed *TFs* were from the *MADS-box* family. *MADS-box TF 5* was majorly expressed in the mutant_14d sample. However, other *MADS-box TFs* were significantly expressed in the two genotypes (Figure 6). Moreover, differential transcriptions of some members of the *NAC*, *SPL*, and *DREB* families were detected in the data of the present study. *MYB*, *HMG*, *PCF*, *HAP3*, *Zinc-finger*, and *PHD-finger* TFs were differentially regulated in the leaves of the two rice genotypes during the grain-filling stage.

### 2.7. DEGs Related to ATPase in the Two Genotypes

In this study, seven DEGs between 0 day and 14 days in the *ospls1* mutant were related to ATPase, whereas one DEG between 0 day and 14 days was detected in the wild type (Supplementary Table S5, Table 5). For the *ospls1* mutant, three DEGs encoding ATP synthase, ATP synthase subunit b, and putative ATPase decreased their transcripts to 1.2–2.2 times on the 14th day of the grain-filling stage. By contrast, two DEGs encoding ATP synthase subunit beta showed a slight increase in transcripts. In addition, the expression levels of two DEGs controlling V-type proton ATPase subunit F were increased in the *ospls1* mutant. However, only one DEG encoding ATP synthase subunit b showed a significant decrease in transcript for the wild type.

### 2.8. DEGs Associated with Hormone Signaling

Except DEGs encoding uncharacterized proteins, 11 hormone-related DEGs between 0 day and 14 days in the *ospls1* mutant were identified, which were involved in the metabolisms of auxin/IAA, cytokinins (CTKs), BRs, and ABA in rice leaves (Supplementary Table S6, Table 6). All DEGs showed varying degrees of decreasing transcription abundance in the *ospls1* mutant during leaf senescence, suggesting the weakened hormone signaling pathways in senescing leaves of the *ospls1* mutant. Particularly, the expression of *brassinazole-resistant 1 homolog 1* (*BZR1*) decreased to nearly 3.3 times in the *ospls1* mutant on the 14th day of the grain-filling stage. However, for the wild type, the DEGs associated with the BR signaling pathway did not show changes in their transcripts. Moreover, two DEGs encoding probable indole-3-acetic acid-amido synthetase GH3.8 and putative ABA-induced protein were also not detected to be differentially expressed in the leaves of the wild type during the grain-filling stage.

### 2.9. DEGs Are Involved in the Antioxidative Metabolism and Cyanide-Resistant Respiration

Some DEGs related to antioxidative metabolism and cyanide-resistant respiration were identified between 0 day and 14 days in the *ospls1* mutant, which were critical for the regulation of eliminating ROS accumulation and alleviating cellular damages during the leaf senescence process. For antioxidative metabolism, eight DEGs showed decreases in the transcripts, whereas five DEGs displayed slight increases in the transcripts for the *ospls1* mutant (Supplementary Table S7, Table 7), suggesting the deficiencies of transcripts of genes associated with ROS elimination. However, most of the antioxidative genes in the wild type did not significantly change their expression abundances, and only two genes showed slightly decreased expressions. In the wild type, the transcription of a DEG encoding catalase isozyme A (CatA) increased by nearly 4.17 times. For cyanide-resistant respiration, three DEGs encoding alternative oxidase in the *ospls1* mutant displayed significantly differential expressions, indicating their responses to cellular oxidative stress.

### 2.10. DEGs Associated with the Regulation of Carbohydrate Metabolism in the ospls1 Mutant

Carbohydrates play important roles in plant development of rice during the grain-filling stage because of their major contribution to grain yield. In this study, we investigated the DEGs associated with carbohydrate metabolism in the two genotypes during the grain-filling stage. As shown in Supplementary Table S8 and Table 8, a total of 16 DEGs related to hexose metabolism were differentially expressed between 0 day and 14 days in the *ospls1* mutant; 11 of them displayed significantly decreased transcriptions, reducing to nearly 0.61 to 2.55 times, and mainly encoded for fructose-1,6-bisphosphatase, fructose-bisphosphate aldolase, glucose-1-phosphate adenylyltransferase, and glucose-6-phosphate 1-dehydrogenase. Only five DEGs showed slightly increasing transcriptions during the leaf senescence stage. The results suggested that the hexose signaling and transformation between monosaccharides, disaccharides, and polysaccharides gradually decreased in the senescing leaves of the *ospls1* mutant. By contrast, eight DEGs between 0 day and 14 days in the *ospls1* mutant were involved in sucrose metabolism. Among them, five DEGs encoding trehalose-6-phosphate synthase isoforms and sucrose synthase significantly improved their transcripts to 1.56 to 2.47 times, and three showed slightly reducing expressions in the *ospls1* mutant. However, for the wild type, only one DEG encoding sucrose synthase was found to enhance its expression level. For starch metabolism, 10 DEGs between 0 day and 14 days in the *ospls1* mutant were found. Among them, seven significantly reduced their expressions on the 14th day of the grain-filling stage, whereas three showed slightly promoted expressions. Notably, the transcriptions of four DEGs encoding UDP-glucose 6-dehydrogenase, soluble starch synthase 1, soluble starch synthase II-2, and phosphorylase decreased by approximately two times as those in the initial stage of grain filling. These results suggested that the physiologic function of starch synthesis for *ospls1* mutant was evidently impaired in the senescing leaves during the grain-filing stage.

### 2.11. DEGs Involved in the Hydrolysis and Autophagy in Senescing Leaves

Autophagy-related protein and proteasome are major responding proteins during plant cell senescence. In this study, four DEGs encoding autophagy-related proteins, including autophagy-related protein 3, 8A, 8B, and 8C, were detected to significantly promote their transcripts in the leaf of the *ospls1* mutant during the grain-filling stage. In addition, 11 DEGs dominating proteasome subunits, mainly proteasome subunit alpha and beta, were found to enhance their expression levels in the *ospls1* mutant during leaf senescence (Supplementary Table S9, Table 9). These results suggested that autophagy-related proteins and proteasomes probably play indispensable regulatory roles in macromolecular hydrolysis and cell apoptosis during early leaf senescence in the *ospls1* mutant. Compared with those in the *ospls1* mutant, the DEGs in the wild type corresponding to autophagy-related proteins and proteasome subunits did not display significantly differential expressions during the grain-filling stage.

## 3. Discussion

### 3.1. Deficiency of OsVHA-A Expression Led to Early Leaf Senescence in the ospls1 Mutant during the Grain-Filling Stage

V-H^+^-ATPase belongs to the highly evolutionarily conserved protein complex and is anchored in the intracellular endomembrane system of tissues. The protein encoded by *OsVHA-A* is considered to be one of the most important components of V-H^+^-ATPase in plants [21]. Several studies have been performed to determine its diverse functions in the developmental processes and stress response. For example, *VHA-A* has been found to be involved in pollen fertility of *Arabidopsis* [21], regulation of stomatal movement in leaf cells of rice under salt stress and osmotic stress conditions [22], and enhanced leaf senescence and seed dormancy in rice [24]. In this study, the *ospls1* mutant exhibited significant decreases in *OsVHA-A* transcripts and V-H^+^-ATPase activity (Figure 3B,C) and striking decreases in soluble sugar and proteins in flag leaves around the 14th day of the grain-filling stage (Figure 2A,B). In a previous study, we discovered that *ospls1* mutant showed a significant decrease in chlorophyll content and apparent increases in MDA level and relative conductivity for flag leaves compared with wild type during the grain-filling stage [25]. Meanwhile, the *ospls1* mutant plant showed sharp decreases in Fv/Fm and *Pn* after the 14th day of grain filling (Figure 2C,D), followed by a rapid increase of lesion mimics and appearance of early leaf senescence during the same period (Figure 1B). The chlorophyll content, membrane ion leakage, and soluble sugar and proteins are used as senescence markers [1]. Therefore, these above results of the *ospls1* mutant indicate obvious physiological characteristics of early leaf senescence and suggest a negative correlation between the *OsVHA-A* expressions and properties of leaf senescence. In other words, less expression of *OsVHA-A* in the *ospls1* mutant inevitably resulted in lower V-H^+^-ATPase activity in flag leaves compared with that in the wild type, which was probably a principal cause of early leaf senescence in the *ospls1* mutant. Yang et al. [24] asserted that numerous transcripts of *OsVHA-A* or high V-H^+^-ATPase activity is required for delaying leaf senescence in rice, which provided evidence to support our conclusion in the opposite way. Moreover, the significant decreases in available panicles per plant and seed-setting rate were observed in the *ospls1* mutant, which were probably ascribed to the reduced carbohydrate translocation from source leaves owing to early senescence, subsequently leading to marked decreases in 1000-grain weight, yield per plant, and harvest index (Table 1).

### 3.2. TFs Are Highly Enriched during Leaf Senescence

TFs are proteins that bind to the cis-regulatory DNA elements in the promoter of genes to activate or repress their expressions [26]. Previous studies have proved the crucial role of TFs in plant leaf senescence [27]. In the present study, 81 DEGs between 0 day and 14 days in the two genotypes were found to encode TFs (Supplementary Table S4, Figure 6). Among these TFs, the top two TF families identified were Homeobox-ZIP and WRKY, followed by the AUX-IAA and MADS-box families. Homeobox-ZIP TFs have been found to participate in ET and ABA biosynthesis during senescence by interacting with aminocyclopropane-1-carboxylate oxidase gene [28,29]. WRKY TFs have been shown to be involved in the crosstalk between the SA and jasmonic acid (JA) signaling pathways and play a central role in mediating negative crosstalk between pathogen resistance and leaf senescence by functioning in the defense signaling pathways of mitogen-activated protein kinases [30,31]. MADS-box genes have been identified to play important biological roles in flower and vegetative tissue development, senescence, and seed dormancy, such as *SlCMB1* in tomato, which interacts with ripening-related genes (*SIMADS-RIN*, *SIMADS1*, *SIAP2a*, and *TAGL1*) to regulate ET production and carotenoid accumulation during tissue ripening and senescence [32]. AUX-IAA TFs function as transcriptional activators that dominate gene expression in auxin signaling transduction to mediate auxin homeostasis, subsequently affecting the process of leaf senescence in a precise manner in the downstream of the pathway [33,34]. In the present study, WRKY TFs 42, 49, and 70 and MADS-box TF 5 were detected to be specifically enriched in senescing leaves, thereby suggesting that these TFs are probably involved in the process of leaf senescence. Furthermore, the transcriptions of many WRKY genes were induced by H_2_O_2_, supporting the involvement of WRKY TFs in leaf senescence [35]. However, it is also considered a striking functional overlap with the senescence-associated TFs [36], and the present study has provided fundamental clues on TF involvement in the regulation of senescence; further functional studies of senescence-associated TFs are thus imperative.

### 3.3. Regulation of Antioxidative System during Early Leaf Senescence

ROS and the antioxidative system play important regulatory roles in tissue development, senescence, and stress response in plants. Under normal conditions, the production and elimination of ROS in plant cell compartments are in balance in virtue of the activation of antioxidant enzymes, including superoxide dismutase, catalases, peroxidases, and thioredoxin reductase (TRC). However, abiotic or biotic stresses often cause a serious imbalance in any cell compartment because of increasing ROS generation or decreasing antioxidative capacity [37]. Leaf senescence is accompanied by the accumulation of ROS in deteriorating tissues; meanwhile, a loss in activities of antioxidant enzymes occurs during the progression of senescence [38]. The increasing ROS accumulation in turn results in enhanced leaf senescence [25]. In the present study, 16 DEGs encoding antioxidant enzymes committed to ROS levels were significantly enriched (Table 7). Among them, six DEGs encoded SOD, which catalyze the dismutation of O_2_^−^ to H_2_O_2_. Among these six SOD genes, four DEGs significantly reduced their expression in the *ospls1* mutant, and the other two DEGs slightly enhanced their transcriptions, suggesting the weakened elimination capacity for O_2_^−^ through dismutation reaction in *ospls1* mutant, which led to the accumulation of O_2_^−^ in *ospls1* mutant leaves [39]. Moreover, three DEGs encoded catalase isozymes, which convert H_2_O_2_ to water and molecular oxygen. The expression levels of DEGs for *CatA* and *CatB* were significantly enhanced in the *ospls1* mutant. Our previous study revealed that the expression levels of *CatA* and *CatB* were promoted by H_2_O_2_ in senescing leaves, which were conducive to alleviate oxidative damages to leaf cells in the early stage of senescence [25]. By contrast, four decreased transcripts of DEGs identified in the *ospls1* mutant were involved in the thioredoxin (Trx) system, in which TRC is the unique enzyme known to catalyze the reduction of Trx. Together with Trx and NADPH, the Trx system can form reduced disulfide bonds and plays an important role in defense against oxidative damage due to oxygen metabolism in plant cells [40]. Carrillo et al. [41] found that the decrease in ATP synthase activity in *Arabidopsis* mutants lacking *TRC* led to a buildup of the proton motive force with subsequent activation of non-photochemical quenching and downregulation of linear electron flow and concluded that *TRC* provides redox modulation at light stress using the relatively oxidizing substrate NADPH. Therefore, decreased *OsVHA-A* transcripts and V-H^+^-ATPase (Figure 3) in the *ospls1* mutant were probably involved in the weakened redox modulation through depressing *TRC* transcription. Meanwhile, the increasing transcriptions of DEGs encoding alternative oxidative (Table 7) in *ospls1* mutant probably contributed to an increased respiration rate and consume excessive electron flow, which perhaps exacerbated the intracellular stresses and in turn aggravated leaf senescence. Moreover, the enhanced cyanide-resistant respiration dominated by an alternative oxidative exacerbated macromolecular hydrolysis and organelle apoptosis in the senescing leaf tissues was confirmed by the results of increased expressions of 15 DEGs encoding proteasome subunits and autophagy-related proteins in the present study (Table 9).

### 3.4. Involvement of Hormone Signals during Early Leaf Senescence

Plant hormones have been thought to play crucial regulatory roles in either delaying or promoting leaf senescence; for instance, auxin and CTKs are approved to delay leaf senescence, whereas ABA, ET, and BRs are thought to accelerate leaf senescence [42,43]. In this study, 11 downregulated DEGs between 0 day and 14 days were enriched in hormone signaling pathways during leaf senescence of the *ospls1* mutant, which were involved in the regulation of auxin, CTKs, BRs, and ABA (Table 6). As an important stress hormone, ABA plays versatile functions in the regulation of environmental stresses and tissue development [44]. In plants, ABA levels are the result of a balance between biosynthesis and catabolism [45]. Zeaxanthin epoxidase, which converts zeaxanthin into violaxanthin, has been shown to play an important role in ABA biosynthesis, whereas ABA 8′-hydroxylase is the key enzyme involved in the oxidative degradation of ABA [45,46]. In *Arabidopsis*, the genes associated with ABA biosynthesis were downregulated during senescence, and many genes involved in ABA catabolism were significantly upregulated [47]. In this study, one DEG encoding zeaxanthin epoxidase decreased its transcript to around 0.63 times in the senescing leaves of the *ospls1* mutant (Table 6), indicating the existence of slightly weakened ABA biosynthesis in senescing leaves. By contrast, two DEGs encoding for ABA 8′-hydroxylase 2 and ABA 8′-hydroxylase 3 showed decreasing transcripts by nearly 1.4 and 1.1 times (Table 6), thereby implying the severely impaired catabolism of ABA, which was probably one reason why excess ABA accumulated in the senescing leaves [39]. In addition, a key ABA signal transduction-related DEG encoding ABA-induced protein was enriched in senescing leaves, which suggested that ABA signaling transduction is activated during leaf senescence. Therefore, ABA is regarded as a hormonal trigger or accelerator of leaf senescence by promoting ABA levels and enhancing ABA signaling transduction [36].

BRs are a group of important hormones that play pivotal roles in a variety of plant growth and developmental processes, including leaf senescence. The BR signaling pathway plays a central role in the involvement of BRs on leaf senescence [48]. In this study, two DEGs encoding BR insensitive 1 (BRI1) kinase inhibitor 1 (BKI1) and BZR1 were enriched in the BR signaling pathway, which showed significantly decreasing transcripts by nearly 1.47 and 3.31 times in senescing leaves of *ospls1* mutant (Table 6). BKI1 functions as a negative regulator of BRI1. In the presence of BRs, BKI1 phosphorylated by BRI1 delocalizes from the plasma membrane into the cytoplasm. The plasma membrane-dissociated and phosphorylated BKI1 plays positive roles in the BR signaling pathway. BZR1 performs an indispensable function in response to BRs through ATP-dependent phosphorylation or dephosphorylation catalyzed by protein phosphatase [49]. However, the decreasing transcripts associated with ATPase were observed in senescing leaves of the *ospls1* mutant (Table 5), which suggested that ATP synthesis catalyzed by ATPase was prevented in the *ospls1* mutant, thereby probably resulting in the deficiency of ATP for ATP-dependent phosphorylation or dephosphorylation during leaf senescence. Moreover, previous studies have reported that BR biosynthesis and central transmitters of the BR signaling pathway from the receptors to the target genes did not increase during leaf senescence [36]. Therefore, the depressed expressions of BKI1 and BZR1 in senescing leaves probably severely counteracted the BR signaling pathway in *ospls1* mutant because of its low V-H^+^-ATPase activity in the intracellular membrane of various organelles. However, the relationships between the BR signaling pathway and V-H^+^-ATPase remain to be investigated.

Auxin and CTKs play critical regulatory roles in delaying leaf senescence [43]. Previous studies have reported that indole acetic acid biosynthetic genes showed significant changes during age-dependent leaf senescence [36]. In this study, one DEG encoding indole-3-acetic acid-amido synthetase GH3.8 displayed a significant decline in transcript abundance during leaf senescence of the *ospls1* mutant. The DEG encoding AUX1-like permease, which is one of the auxin efflux carrier components, showed significantly decreasing transcription. Moreover, two DEGs encoding auxin-responsive protein IAA5 and auxin-responsive protein IAA13 were involved in the auxin signal transduction and exhibited decreased expression levels during leaf senescence (Table 6). These results suggested that changes in auxin synthesis, signaling transduction, and transport are probably important in modulating leaf senescence. In addition, a few studies reported that CTKs and the CTK signaling pathway are involved in an earlier event during the senescence stage [50]. However, in our study, only one DEG encoding type A response regulator1 showed a slight decline in the transcript for the two rice genotypes, suggesting that CTKs and the corresponding signaling pathway perhaps are not involved in the regulation of early leaf senescence. This finding is in agreement with the conclusion on leaf senescence in cotton [36].

### 3.5. Relationship between Carbohydrates Translocation and Leaf Senescence

Rice yield mainly depends on assimilate translocation from source leaves to developing grains during the grain-filling stage [51]. An early occurrence of leaf senescence caused by intrinsic genetic factors or by adverse stresses results in a drop in photosynthesis and curtails the supply of photoassimilates from the leaves [7]. In this study, the decreases in *Pn* (Figure 2D) and soluble sugar (Figure 2A) were observed in the leaves of the *ospls1* mutant, which probably resulted in the appearance of sugar starvation in senescing leaves. There is a large body of evidence to support that sugar starvation enhances the progression of leaf senescence [52]. In this study, many DEGs involved in hexose, sucrose, and starch metabolism were enriched in the mutant during leaf senescence. For hexose metabolism, some DEGs encoding fructose-1,6-bisphosphatase, fructose-bisphosphate aldolase, glucose-1-phosphate adenylyltransferase, and glucose-6-phosphate 1-dehydrogenase showed significant decreases in transcripts for *ospls1* mutant (Table 8), suggesting that hexokinase-dependent glucose and sucrose signaling pathways appeared to be inactive in senescing leaves owing to the decreases in *OsVHA-A* transcript and V-H^+^-ATPase activity. By contrast, assimilate remobilization is enhanced once the leaf senescence is initiated in cereals [8], and sucrose is a major assimilate translocated from source leaves to developing grains [53]. In the present study, five DEGs encoding trehalose-6-phosphate synthase and sucrose synthase displayed significantly increasing expression levels in the *ospls1* mutant, whereas DEGs encoding sucrose-phosphate synthase and beta-fructofuranosidase exhibited slightly decreasing expression levels (Table 8), which indicated that sucrose cleavage catalyzed by trehalose-6-phosphate synthase and sucrose synthase was enhanced and that sucrose synthesis, dominated by sucrose-phosphate synthase and beta-fructofuranosidase, was weakened during leaf senescence. Starch is a major carbohydrate remobilized in source leaves of rice plant during the grain-filling stage. Along with the progression of leaf senescence, starch stored in source leaves before heading is remobilized and translocated to the developing grains during the grain-filling stage, subsequently contributing to the final yield [54]. In this study, the DEGs between 0 day and 14 days involved in starch synthesis showed significant decreases in transcripts for leaves of the *ospls1* mutant, and DEGs encoding beta-amylases exhibited increasing expression levels, which clearly proved that starch remobilization was enhanced in senescing leaves. Moreover, sucrose translocation and starch remobilization from source leaves to developing grains also exacerbated the phenomenon of sugar starvation in senescing leaves, which in turn accelerated the progression of leaf senescence.

## 4. Materials and Methods

### 4.1. Plant Materials and Growth Conditions

The *ospls1* mutant rice with early senescence phenotype was obtained from the mature seeds of gramma-irradiated cultivated rice Fu142 (*Oryza sativa* L. ssp. *indica*). Stable phenotype selection was conducted from the M2 to M8 generations through successive self-pollination. The *ospls1* mutant plant presented normal phenotypic appearance between the seedling and tillering stages. Until the late tillering stage, brown lesions began to appear on the tips of the lower leaves of the *ospls1* mutant rice plant, and gradually became exacerbated and covered the whole leaf blade. However, the topmost two fully expanded leaves and central leaf of rice plant kept their normal green appearance at the same period. After the flowering stage, the topmost flag leaf of the *ospls1* mutant plant began to show senescence symptoms, and the brown lesions gradually spread from the tip down to the whole leaf blade during the grain-filling stage. The flag leaf became completely withered about 3 weeks after anthesis. By contrast, the wild type still retained its normal green appearance during the whole same period (Figure 1A). Moreover, a cytosine deletion in the gene encoding V-H^+^-ATPase subunit A has been identified to underlie the early senescence phenotype in the *ospls1* mutant [24].

The field experiment was performed at the experimental field of Fujian Agriculture and Forestry University in Fuzhou, China. Rice seedlings were cultivated in April 15 and then transplanted May 14. The field plots were arranged as a random design, followed by three replications for each genotype. Each replication was set as 10 × 12 rows, and planting distance was performed according to 18 cm × 18 cm with one seedling for each hill. The field experiment was managed according to the local planting practices, and the soil property was the periodically waterlogged paddy soil, with 1.69 g/kg total N, 24.5 mg/kg available P, and 103.7 mg/kg exchangeable K. The samples were collected during the grain-filling stage. Rice plants with uniform anthesis date were randomly selected and tagged at the full heading day. The chlorophyll maximal quantum yield of PSII photochemistry (Fv/Fm) and net photosynthetic rate (*Pn*) were determined over 7-day intervals during the grain-filling stage. The flag leaves of tagged plants were sampled over 7-day intervals during the grain-filling stage and were used to determinate the physiological parameters. In addition, the whole flag leaf of each genotype was sampled on day 0 and 14 of grain-filling stage and immediately frozen in liquid nitrogen and kept at −80 °C for RNA extraction and RNA-seq. Three independent whole flag leaves of each rice plant were treated as the biological replications for each genotype on day 0 and 14 of grain-filling stage, a total of 12 flag leaf samples were collected in this study. At maturity, 15 tagged plants were harvested from each plot to investigate the agronomic traits and yield characteristics.

### 4.2. Measurement of Fv/Fv, Pn, Soluble Sugar and Protein, and V-H^+^-ATPase Activity

Before Fv/Fm measurement, whole rice plant was dark-adapted for 20 min, and chlorophyll Fv/Fm was measured using a steady-state gas-exchange system with an integrated fluorescence chamber head (LI-6400-40, LI-COR, Lincoln, NE, USA). Fv/Fm value was calculated as Fv/Fm = (Fm−F_0_)/Fm [55]. *Pn* was determined using the LI-6400 portable photosynthesis system (LI-COR, Lincoln, NE, USA) under a 2000 μmol·m^−2^·s^−1^ of photosynthetic photon flux density (PPFD), 370 μmol·mol^−1^ of CO_2_ concentration and a 1.3–1.6 kPa leaf-to-air vapor-pressure difference. During measurement, leaf temperatures were maintained at 30 °C. All of the measurements were conducted at 9:00–11:00 a.m. The soluble sugar was determined using the anthrone-H_2_SO_4_ colorimetry method [56], and soluble protein in fresh leaves was determined by the method of Bradford [57]. The activity of V-H^+^-ATPase was evaluated by determining the release of phosphate (Pi) and expressed in μmol Pi·mg^−1^ protein·h^−1^. Membrane proteins were extracted from fresh samples, and 10 μg of microsomal membranes was incubated for 40 min at 28 °C. Afterward, 40 mM of citric acid was added to impede the reaction. Meanwhile, 10 μg of bovine serum albumin was used as a reference. The V-H^+^-ATPase activity was calculated, as described by Zhang et al. [22].

### 4.3. RNA Extraction and RNA-seq

For RNA-seq, RNA extraction was performed according to the manufacturer’s protocol of the RNAprep Pure Plant kit (TianGen Biotech Co., Ltd., Beijing, China). RNA purity and integrality were checked before cDNA library construction. mRNA was enriched from the total RNA through the magnetic beads with oligo (dT). Afterward, mRNA was randomly broken into fragments. Catalyzed by reverse transcriptase, double-stranded cDNA (ds-cDNA) was synthesized through mRNA by combining with random primers. After purifying ds-cDNA, the adapters and poly(A) tails were ligated at both ends of ds-cDNA. With the adapter sequences, ds-cDNA fragments were amplified by PCR reaction. The cDNA libraries were then ready for sequencing. After accurate ds-cDNA quantification, ds-cDNA library sequencing was conducted using the Illumina HiSeq2000 platform (Illumina, San Diego, CA, USA).

### 4.4. Bioinformatics Analysis of RNA-seq Data and Identification of DGEs

The raw sequence data were generated through the Illumina data processing pipeline (version 1.8). The clean data were obtained by removing adaptor sequences and low-quality reads (reads with more than 50% of bases having *Q* value ≤20 or an ambiguous sequence content exceeding 10%). A reference genome-based transcriptome analysis strategy was employed to process the high-quality sequence data. The rice reference genome was downloaded from the Genomics website (ftp://ftp.ensemblgenomes.org/pub/release-23/plants/fasta/oryza_indica/dna/). TopHat was used to align the high-quality reads to the rice reference genome, and then mapping results were built to identify known and novel transcripts.

The expression levels of genes in all samples were evaluated by quantifying the count of mapped reads. The FPKM method was employed to normalize gene expression counts for the sequence. The expression ratios between different sample groups (Mutant_14d vs. Mutant_0d, Wild_14d vs. Wild_04, Mutant_0d vs. Wild_0d, and Mutant_14d vs. Wild_14d) were calculated as fold changes. Significant DEGs were identified by the DESeq software package, according to |log_2_ (Fold Change)| > 1 and adjusted *p*-value (padj) < 0.05 [58].

### 4.5. qRT-PCR Validation

The RNA for RNA-seq was also used for qRT-PCR assays to validate the reliability of RNA-Seq data. Approximately 1 µg of total RNA was treated with RNase-free DNaseI to eliminate genomic DNA pollution, and then cDNA was synthesized in a 50 µL reaction with an oligo (dT) primer by using the ReverTra Ace qPCR RT kit (Toyobo, Osaka, Japan). qRT-PCR was performed on a CFX96 system machine (Bio-Rad, Hercules, CA, USA) according to the protocols, with the 20 µL reaction solution containing 10 µL of SYBR, 1 µL of cDNA, 1.6 µL of 10 mM primer, and 7.4 µL of H_2_O. The specific primers of genes chosen were designed by Primer-BLAST (http://www.ncbi.nlm.nih.gov/tools/primer-blast/) as listed in Supplementary Table S1. To normalize data, the amplification of *ACTIN-1* sequence was performed as an endogenous reference. The relative expressions of genes were calculated by the comparative *C*_t_ method [59].

### 4.6. Functional Annotation, Gene Ontology (GO) Enrichment, and KEGG Analysis of DEGs

DEGs were submitted to GO and KEGG enrichment (http://www.genome.jp/kegg/) analysis to annotate their biological function and significantly metabolic pathways. The submission of DEGs to GO enrichment was performed using GOseq method based on Wallenius non-central hypergeometric distribution [60]. The GO distributions associated with identified DEGs were achieved according to three levels: biological process, molecular function, and cellular component. The KOBAS 2.0 software was then used to identify the significant pathways of DEGs in KEGG enrichment. Pathways with corrected *p*-value < 0.05 were considered statistically significant.

## 5. Conclusions

Taken together, our results showed that the *ospls1* mutant rice underwent early leaf senescence in comparison with its wild type during the grain-filling stage. Physiological and agronomic analysis showed that the *ospls1* mutant displayed significant decreases in soluble sugar and protein, *Pn*, and yield traits, accompanied by decreases in *OsVHA-A* transcript and V-H^+^-ATPase activity. RNA-Seq results suggested that approximately 4827 DEGs between 0 day and 14 days in the *ospls1* mutant were identified and analyzed for their potential roles in leaf senescence using GO enrichment and KOBAS analysis. Moreover, 81 differentially expressed TFs between 0 day and 14 days were identified, and some of them are critical for the regulation of plant senescence. Eleven hormones associated with DEGs were enriched in the auxin, CTK, BR, and ABA pathways, indicating that hormone-signaling pathways were involved in leaf senescence for the *ospls1* mutant. In addition, many antioxidative and carbohydrate metabolism-related genes were found to be differentially expressed in *ospls1* mutant leaves, suggesting that these genes probably play responding and regulatory roles in leaf senescence. Therefore, the present study revealed a large number of candidate genes and pathways that perhaps play important roles in leaf senescence of rice. Meanwhile, the interaction relationships between key candidate genes and *OsVHA-A* will be feasible in future research, which will be helpful for understanding the molecular mechanisms involved in the early leaf senescence of rice.

## Figures and Tables

**Table 1:** Variation of important agronomic traits between *ospls1* mutant and wild type.

Traits	2015			2016		
	Wild Type	Mutant	Different Significance	Wild Type	Mutant	Different Significance
Growth duration	88.1 ± 2.1	86.6 ± 2.4	ns	89.6 ± 3.1	88.6 ± 2.4	ns
Plant height (cm)	109.3 ± 3.3	92.4 ± 2.2	*	110.8 ± 3.8	94.3 ± 3.6	*
Available panicle number	11.7 ± 3.8	6.3 ± 2.4	**	11.1 ± 2.4	5.6 ± 0.8	**
Grain shape	2.64 ± 0.02	2.58 ± 0.1	ns	2.61 ± 0.05	2.54 ± 0.08	ns
Seed-setting rate (%)	84.5 ± 3.9	41.8 ± 6.8	**	82.2 ± 6.1	36.6 ±4.3	**
1000-grain weight (g)	23.37 ± 1.1	17.62 ± 1.6	**	23.14 ± 0.6	18.1 ± 1.3	**
Yield per plant (g)	53.0 ± 7.4	4.1 ± 1.8	**	48.6 ± 9.5	3.8 ± 0.6	**
Harvest index	0.44 ± 0.03	0.15 ± 0.03	**	0.46 ± 0.02	0.19 ± 0.03	**

Grain shape was determined by the ratio of grain length to width. * and ** represent significant differences at 0.05 and 0.01 probability levels, respectively. Growth duration means the days from sowing to the heading stage, and ns represents no significant difference.

**Table 2:** Overview of the RNA-sequencing reads generated from each sample.

Sample	Raw Reads_count	Clean Reads_count	Total Mapped Reads_count	Mapped %	Uniquely Mapped Reads_count	Uniquely Mapped %
Mutant_0d1	50,966,886	49,401,112	39,881,950	80.73	38,567,821	78.07
Mutant_0d2	60,634,598	58,467,030	47,637,811	81.48	46,087,811	78.83
Mutant_0d3	51,135,352	49,444,022	39,862,112	80.62	38,601,162	78.07
Wild_0d1	50,698,138	48,773,770	39,684,358	81.36	38,377,545	78.68
Wild_0d2	46,710,304	44,703,316	36,126,426	80.81	34,969,393	78.23
Wild_0d3	54,065,590	51,885,202	42,252,458	81.43	40,807,059	78.65
Mutant_14d1	50,030,824	48,186,294	37,512,316	77.85	36,507,456	75.76
Mutant_14d2	45,736,398	43,690,018	30,590,126	70.02	29,775,623	68.15
Mutant_14d3	49,065,914	47,170,682	36,361,257	77.08	35,377,483	75.00
Wild_14d1	62,344,682	60,065,524	48,768,418	81.19	47,309,394	78.76
Wild_14d2	54,823,378	52,520,882	42,248,041	80.44	40,998,600	78.06
Wild_14d3	48,894,006	46,946,312	38,265,388	81.51	37,104,074	79.04

The replications are defined as 1, 2, and 3, respectively.

**Table 3:** DEGs identified from four different comparisons. DEGs: differentially expressed genes.

DEG Set	DEGs	Up-Regulated	Down-Regulated
Mutant_14d vs. Mutant_0d	11,504	5312	6192
Wild_14d vs. Wild_0d	3419	1636	1783
Mutant_0d vs. Wild_0d	5215	3093	2122
Mutant_14d vs. Wild_14d	9625	4816	4809

**Table 4:** Significantly enriched pathways in the *OsVHA-A* mutant and wild type by Kyoto Encyclopedia of Genes and Genomes (KEGG) Orthology Based Annotation System (KOBAS) during leaf senescence (Corrected *p*-value < 0.05).

**KEEG ID**	**KEEG Pathway (Down-Regulated)**	**Mutant**			**Wild Type**		
		**Gene Number**	**Background Number**	**Corrected *p*-Value**	**Gene Number**	**Background Number**	**Corrected *p*-Value**
osa01110	Biosynthesis of secondary metabolites	278	779	0.0028	82	779	0.0222
osa00710	Carbon fixation in photosynthetic organisms	41	77	0.0285			
osa00195	Photosynthesis	40	79	0.0462			
osa00910	Nitrogen metabolism				10	27	0.0105
osa00906	Carotenoid biosynthesis				8	26	0.0467
**KEEG ID**	**KEEG Pathway (Up-Regulated)**	**Mutant**			**Wild Type**		
		**Gene Number**	**Background Number**	**Corrected *p*-Value**	**Gene Number**	**Background Number**	**Corrected *p*-Value**
osa04626	Plant-pathogen interaction				29	130	0.0012
osa00071	Fatty acid degradation				15	41	0.0012
osa00941	Flavonoid biosynthesis				8	19	0.0305
osa01110	Biosynthesis of secondary metabolites				95	779	0.0305
osa00592	alpha-Linolenic acid metabolism				10	33	0.0305
osa00520	Amino sugar and nucleotide sugar metabolism				20	103	0.0305

**Table 5:** Genotypic differences in expressions of DEGs associated with ATPase between the initial and 14th day of the grain-filling stage (padj < 0.05).

Gene ID	Log_2_ (Fold Change 14d/0d)		Description
	Mutant	Wild Type	
BGIOSGA009058	−1.22		ATP synthase
BGIOSGA031512	−2.22	−1.03	ATP synthase subunit b, chloroplastic
BGIOSGA004222	0.32		ATP synthase subunit beta
BGIOSGA017602	0.38		ATP synthase subunit beta
Novel01284	−1.99		putative ATPase
BGIOSGA009328	0.59		V-type proton ATPase subunit F
BGIOSGA015836	0.61		V-type proton ATPase subunit F

**Table 6:** Genotypic differences in expressions of DEGs encoding proteins associated with hormone signaling pathways between the initial and 14th day of the grain-filling stage (padj < 0.05). CTKs: cytokinins; BRs: brassinosteroids; ABA: abscisic acid; and BRI1: Brassinosteroid insensitive 1.

Group	Gene ID	Log_2_ (Fold Change 14d/0d)		Description
		Mutant	Wild Type	
Auxin/IAA	BGIOSGA004190	−0.81	−0.77	Auxin-responsive protein IAA5
	BGIOSGA009837	−1.07	−1.00	Auxin-responsive protein IAA13
	BGIOSGA023979	−1.24		Probable indole-3-acetic acid-amido synthetase GH3.8
	BGIOSGA004826	−1.22	−1.13	Putative AUX1-like permease
CTKs	BGIOSGA015027	−0.76	−1.25	Type A response regulator 1
BRs	BGIOSGA029632	−1.47		Probable BRI1 kinase inhibitor 1
	Novel00067	−3.31		Brassinazole-resistant 1 homolog 1
ABA	Novel01323	−0.78		Putative abscisic acid-induced protein
	BGIOSGA026823	−1.39	−1.04	Abscisic acid 8′-hydroxylase 2
	BGIOSGA029635	−1.13	−1.14	Abscisic acid 8′-hydroxylase 3
	BGIOSGA016502	−0.63	−0.67	Zeaxanthin epoxidase, chloroplastic

**Table 7:** Genotypic differences in expressions of DEGs encoding proteins involved in antioxidative metabolism and cyanide-resistant respiration between the initial and 14th day of the grain-filling stage (padj < 0.05).

Group	Gene ID	Log_2_ (Fold Change 14d/0d)		Description
		Mutant	Wild Type	
Anti-oxidative metabolism	BGIOSGA011520	−1.37	−0.70	Catalase
	BGIOSGA007252	2.41	4.17	Catalase isozyme A
	BGIOSGA023636	1.64		Catalase isozyme B
	BGIOSGA019625	0.52		Superoxide dismutase
	BGIOSGA022060	−0.96		Superoxide dismutase
	BGIOSGA022277	−0.77		Superoxide dismutase
	BGIOSGA029201	−0.42		Superoxide dismutase [Cu-Zn]
	BGIOSGA025399	−0.84		Superoxide dismutase [Cu-Zn]
	BGIOSGA023756	0.81		Superoxide dismutase [Cu-Zn]
	BGIOSGA020152	−0.38		Thioredoxin
	BGIOSGA024701	0.34	−0.74	Thioredoxin H-type
	BGIOSGA021328	−0.54	−1.01	Thioredoxin reductase
	BGIOSGA026313	−0.61		Thioredoxin reductase
Cyanide-resistant respiration	BGIOSGA014421	1.28	1.54	Alternative oxidase
	BGIOSGA014422	1.36		Alternative oxidase
	BGIOSGA005788	−0.95		Alternative oxidase

**Table 8:** Genotypic differences in expressions of DEGs associated with carbohydrate metabolism between the initial and 14th day of the grain-filling stage (padj < 0.05).

Group	Gene ID	Log_2_ (Fold Change 14d/0d)		Description
		Mutant	Wild Type	
Hexose	BGIOSGA000339	−0.64	−0.68	Fructokinase-1
	BGIOSGA004865	−0.93		Fructose-1,6-bisphosphatase, cytosolic
	BGIOSGA027739	−1.95		Fructose-bisphosphate aldolase
	BGIOSGA034421	−1.88		Fructose-bisphosphate aldolase
	BGIOSGA019844	0.54		Fructose-bisphosphate aldolase
	BGIOSGA023247	−2.52		Fructose-bisphosphate aldolase
	BGIOSGA017490	−0.63		Glucose-1-phosphate adenylyltransferase
	BGIOSGA027135	−0.67		Glucose-1-phosphate adenylyltransferase
	BGIOSGA009855	−0.75		Glucose-1-phosphate adenylyltransferase
	BGIOSGA030039	−0.95		Glucose-1-phosphate adenylyltransferase
	BGIOSGA024440	0.44		Glucose-6-phosphate 1-dehydrogenase
	BGIOSGA012859	−1.66	0.76	Glucose-6-phosphate 1-dehydrogenase
	BGIOSGA010851	1.09	−1.43	Glucose-6-phosphate 1-dehydrogenase
	BGIOSGA016632	−0.61	1.29	Glucose-6-phosphate 1-dehydrogenase
	BGIOSGA033719	1.41	1.42	Mannose-6-phosphate isomerase
	BGIOSGA017373	0.53		Phosphomannomutase
Sucrose	BGIOSGA000239	−0.69		Probable sucrose-phosphate synthase 1
	BGIOSGA011194	−0.67		Trehalose-6-phosphate synthase 4
	BGIOSGA026976	2.47		Trehalose-6-phosphate synthase 7
	BGIOSGA028759	2.15		Trehalose-6-phosphate synthase 8
	BGIOSGA030372	−0.55		Beta-fructofuranosidase, insoluble isoenzyme 7
	BGIOSGA010570	2.45		Sucrose synthase
	BGIOSGA021739	0.40		Sucrose synthase
	BGIOSGA010770	1.56	0.87	Sucrose synthase
Starch	BGIOSGA013592	−2.13	1.83	UDP-glucose 6-dehydrogenase
	BGIOSGA037342	0.75		UDP-glucose 6-dehydrogenase
	BGIOSGA031231	−0.38		UDP-glucose pyrophosphorylase
	BGIOSGA021860	−2.08	−1.53	Soluble starch synthase 1, chloroplastic
	BGIOSGA005631	−1.96	−1.19	Soluble starch synthase II-2
	BGIOSGA011829	1.46		Beta-amylase
	BGIOSGA033092	0.72		Beta-amylase
	BGIOSGA000478	−0.46		Phosphorylase
	BGIOSGA009780	−2.01	−1.52	Phosphorylase
	BGIOSGA004591	1.05		Pectinesterase

**Table 9:** Genotypic differences in expressions of DEGs involved in the hydrolysis and autophagy between the initial and 14th day of the grain-filling stage (padj < 0.05).

Group	Gene ID	Log_2_ (Fold Change 14d/0d)		Description
		Mutant	Wild Type	
Regulation of autophagy	BGIOSGA002187	1.05		Autophagy-related protein 3
	BGIOSGA024235	1.03		Autophagy-related protein 8A
	BGIOSGA014317	1.02		Autophagy-related protein 8B
	BGIOSGA028123	0.71		Autophagy-related protein 8C
Proteolysis	BGIOSGA004666	0.45		Proteasome subunit alpha type
	BGIOSGA008679	0.44		Proteasome subunit alpha type
	BGIOSGA011334	0.45		Proteasome subunit alpha type
	BGIOSGA017816	0.52		Proteasome subunit alpha type
	BGIOSGA026895	0.75		Proteasome subunit alpha type-2
	BGIOSGA029124	0.43		Proteasome subunit alpha type-7-A
	BGIOSGA005534	0.43		Proteasome subunit beta type
	BGIOSGA021971	0.36		Proteasome subunit beta type
	BGIOSGA019302	0.50		Proteasome subunit beta type
	BGIOSGA029040	1.58		Proteasome subunit beta type
	BGIOSGA029445	0.39		Proteasome subunit beta type

## Supplementary Materials

Supplementary materials can be found at https://www.mdpi.com/1422-0067/20/5/1098/s1.

## Abbreviations

ABA	Abscisic acid
BKI1	Brassinosteroid insensitive 1 kinase inhibitor 1
BRI1	Brassinosteroid insensitive 1
BRs	Brassinosteroids
BZR1	Brassinazole-resistant 1
Cat	Catalase
CTKs	Cytokinins
DEGs	Differentially expressed genes
ET	Ethylene
FPKM	Fragments per kilobase of transcript sequence per million base pairs sequenced
Fv/Fm	Maximal quantum yield of PSII photochemistry
GO	Gene ontology
H_2_O_2_	Hydrogen peroxide
KEGG	Kyoto encyclopedia of genes and genomes
KOBAS	The KEGG Orthology Based Annotation System
MDA	Malondialdehyde
^1^O_2_	Singlet oxygen
O_2_^−^	Superoxide anion radicals
OH∙	Hydroxyl radicals
*ospls1*	Oryza sativa premature leaf senescence 1
Pn	Net photosynthesis rate
PPFD	Photosynthetic photon flux density
qRT-PCR	Quantitative real-time polymerase chain reaction
ROS	Reactive oxygen species
SA	Salicylic acid
SAGs	Senescence-associated genes
TFs	Transcription factors
TRC	Thioredoxin reductase
Trx	Thioredoxin
VHA-A	V-H^+^-ATPase subunit A
