# Supplementary material for: Physiological and Transcriptome Analyses of Early Leaf Senescence for ospls1 Mutant Rice (Oryza sativa L.) during the Grain-Filling Stage
Source: Int J Mol Sci. 2019 Mar 4;20(5):1098. doi: 10.3390/ijms20051098 (PMC6429080; doi:10.3390/ijms20051098)
Supplement: Supplementary file 1 [file ijms-20-01098-s001.zip › ijms-411580 - supplementary/Supplementary_Table_S1.docx]

**TABLE S1 Primers are listed for real-time PCR.**

| Gene ID | Description | Primer sequence (From 5′ to 3′) |
| --- | --- | --- |
| BGIOSGA002291 | Phospholipase D | F: CAGTTTCGTTGGTGGCCTTG  R: CCTTCCAGCCGTGAGTGAAT |
| BGIOSGA004865 | Fructose-1,6-bisphosphatase, cytosolic | F: TGGGAAGTATTGCGTGTGCT  R: TTCCAGGCTGTAACACGTCC |
| BGIOSGA033901 | Auxin response factor 23 | F: TCTTCCATACCTCGCCCTGA  R: ATTCGGACGAAGCCTCTTGG |
| BGIOSGA005667 | Glutamine synthetase | F: AGGAGCCCTGGTACGGTATT  R: GCAAGCCTTGTAGTGGGAGT |
| BGIOSGA009780 | Phosphorylase | F: GTCTAGGCCGGTTAGCTTCC  R: TCCTCCTGACCATCCTTCGT |
| BGIOSGA009837 | Auxin-responsive protein IAA13 | F: TCCGCAGGAACATCATGACC  R: ATCTTCAGGTCCACCTTGCG |
| BGIOSGA010770 | Sucrose synthase | F: TTTGGGCAGTTCCGTTGGAT  R: TAGAAAGCTGGCTGCACGAA |
| BGIOSGA012029 | Ubiquitin carboxyl-terminal hydrolase 26 | F: CAGCTTGCTCGGTTATTCGC  R: GCACAATGGTTCTAGCCCCT |
| BGIOSGA014317 | Autophagy-related protein 8B | F: ATGGCCAAGAGCTCGTTCAA  R: ATCAGCAGGGACCAGGTACT |
| BGIOSGA020152 | Thioredoxin | F: ACGTCGATGAACTGAAGCCT  R: CATAGCACCGACAACCCTGT |
| BGIOSGA025367 | Cytochrome b-c1 complex subunit 7 | F: GACCTGTACGACCCCTACCA  R: TAGCCTCTGAACGGTGTCTG |
| BGIOSGA025399 | Superoxide dismutase [Cu-Zn] | F: CCGGGCCCCATTTTAATCCT  R: ACAACAACTGCCCTTCCCAA |
| BGIOSGA026097 | MADS-box transcription factor 18 | F: CACTAACCGGGAGGAGCAAA  R: TGAGCTTCTGACTCCCCTGA |
| BGIOSGA026823 | Abscisic acid 8'-hydroxylase 2 | F: AAGCTTCTCGAAGCCGTCAA  R: TGATGCGTGAGTGTCATGCT |
| NM_001064815 | Vacuolar H^+^-ATPase subunit A | F: GCTAACACATCCAACATGCC  R: TTATAGCCCATGTCACGGAA |
| X16280 | *Actin* | F: CAGCACATTCCAGCAGATGT  R: TAGGCCGGTTGAAAACTTTG |
