# Supplementary figures and images for: Physiological and Transcriptome Analyses of Early Leaf Senescence for ospls1 Mutant Rice (Oryza sativa L.) during the Grain-Filling Stage
Source: Int J Mol Sci. 2019 Mar 4;20(5):1098. doi: 10.3390/ijms20051098 (PMC6429080; doi:10.3390/ijms20051098)

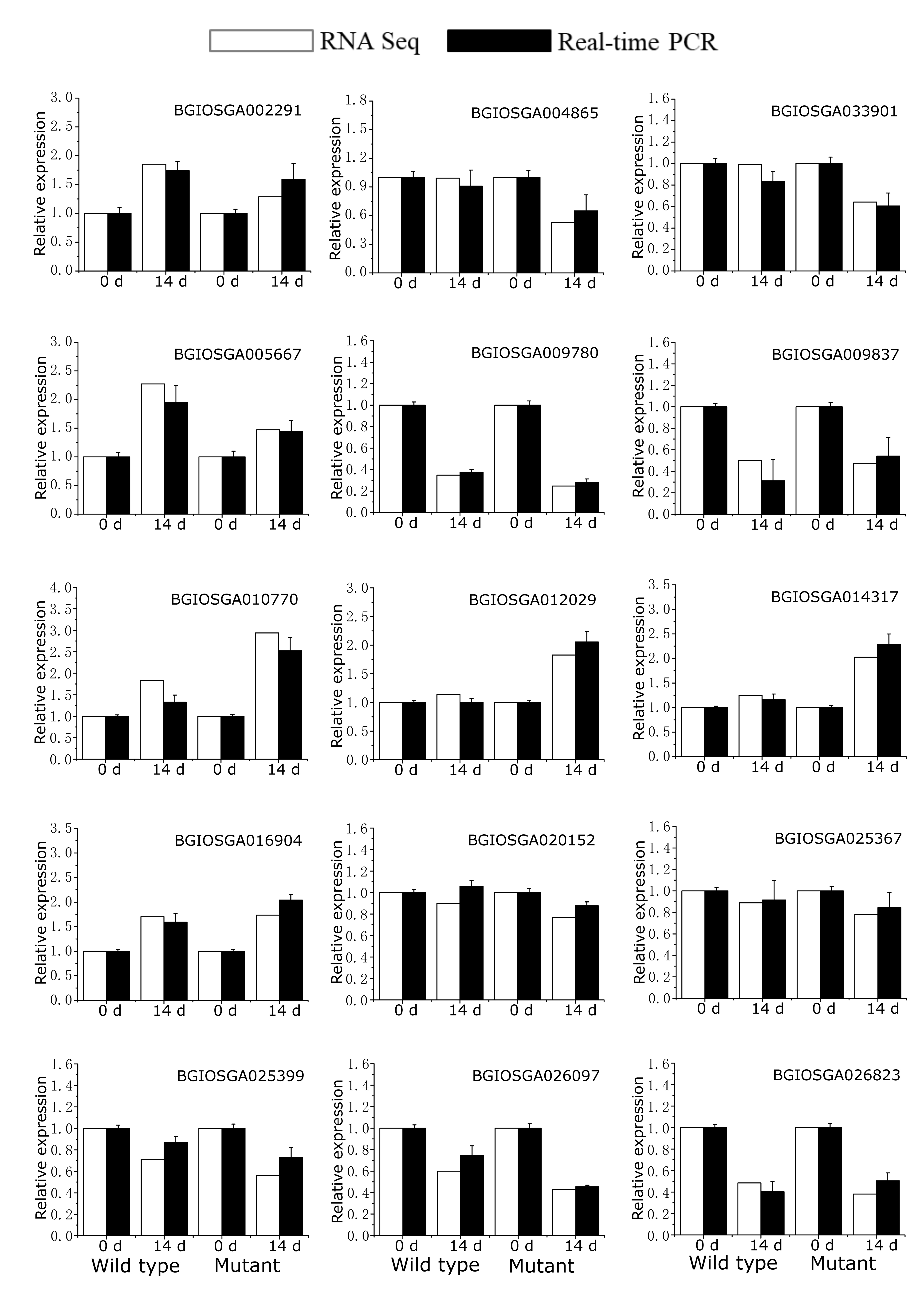

Supplement: Supplementary file 1 [file ijms-20-01098-s001.zip › ijms-411580 - supplementary/ijms-411580-supplementary.jpg]
